# eHealth for Addressing Balance Disorders in the Elderly: Systematic Review

**DOI:** 10.2196/22215

**Published:** 2021-04-28

**Authors:** Andréa G Martins Gaspar, Luís Velez Lapão

**Affiliations:** 1 Hospital Beatriz Ângelo Lisbon Portugal; 2 Global Health and Tropical Medicine Instituto de Higiene e Medicina Tropical Universidade NOVA de Lisboa Lisbon Portugal

**Keywords:** balance disorders, falls, elderly, eHealth, telemedicine

## Abstract

**Background:**

The population is aging on a global scale, triggering vulnerability for chronic multimorbidity, balance disorders, and falls. Falls with injuries are the main cause of accidental death in the elderly population, representing a relevant public health problem. Balance disorder is a major risk factor for falling and represents one of the most frequent reasons for health care demand. The use of information and communication technologies to support distance healthcare (eHealth) represents an opportunity to improve the access and quality of health care services for the elderly. In recent years, several studies have addressed the potential of eHealth devices to assess the balance and risk of falling of elderly people. Remote rehabilitation has also been explored. However, the clinical applicability of these digital solutions for elderly people with balance disorders remains to be studied.

**Objective:**

The aim of this review was to guide the clinical applicability of eHealth devices in providing the screening, assessment, and treatment of elderly people with balance disorders, but without neurological disease.

**Methods:**

A systematic review was performed in accordance with the PRISMA (Preferred Reporting Items for Systematic Reviews and Meta-Analysis) statement. Data were obtained through searching the PubMed, Google Scholar, Embase, and SciELO databases. Only randomized controlled trials (RCTs) or quasiexperimental studies (QESs) published between January 2015 and December 2019 were included. The quality of the evidence to respond to the research question was assessed using Joanna Briggs Institute (JBI) Critical Appraisal for RCTs and the JBI Critical Appraisal Checklist for QESs. RCTs were assessed using the Cochrane risk of bias tool. We provide a narrative synthesis of the main outcomes from the included studies.

**Results:**

Among 1030 unduplicated articles retrieved, 21 articles were included in this review. Twelve studies explored different technology devices to obtain data about balance and risk of falling. Nine studies focused on different types of balance exercise training. A wide range of clinical tests, functional scales, classifications of faller participants, sensor-based tasks, intervention protocols, and follow-up times were used. Only one study described the clinical conditions of the participants. Instrumental tests of the inner ear were neither used as the gold-standard test nor performed in pre and postrehabilitation assessments.

**Conclusions:**

eHealth has potential for providing additional health care to elderly people with balance disorder and risk of falling. In the included literature, the heterogeneity of populations under study, methodologies, eHealth devices, and time of follow-up did not allow for clear comparison to guide proper clinical applicability. This suggests that more rigorous studies are needed.

## Introduction

### Background

#### Aging and Balance Disorders

The improvement of health conditions and the increase in life expectancy have led to an aging global population, although this is not always accompanied by an increase in healthy life years [1-4].

Aging is associated with functional deterioration, including in the peripheral sensory structures, thereby affecting vision, hearing, and balance [5,6]. Additionally, elderly individuals are more likely to suffer from multiple chronic conditions, which often leads to frailty with risk of falls [1-4]. Falls in elderly people represent a serious public health problem as the main cause of accidental death in this population. The risk of falling increases with age [1,7-9]. Each year, approximately one in every three elderly people experiences a serious fall. Moreover, falling can lead to deterioration of the quality of life, anxiety, depression, restriction in daily activities, decreased mobility, social isolation, increased consumption of medications, and increased dependence on medical services and informal caregivers [1,2].

Several causes of falls in the elderly population have been identified, including age, environmental factors (eg, wet paths), inappropriate clothing and shoes, incorrect behavior (eg, climbing chairs), excessive alcohol consumption, inadequate use of medications, deteriorating chronic illness, and balance disorders [1,6,10].

Various clinical conditions are associated with balance disorders in elderly people, including age-related decline in balance function (prebyvestibulopathy); medications; and cardiovascular, metabolic, musculoskeletal, neurologic, and otologic diseases [5,6].

Although dizziness and vertigo are recognized as significant factors increasing the risk of falling and are common symptoms among the elderly, epidemiological studies have revealed large variability in the prevalence of balance disorders in this population [11-13]. It is estimated that at least 30% of individuals above 60 years old suffer from vertigo and dizziness, increasing to 50% for those above 85 years old [13]. According to the 2008 National Health Interview Survey, 33 million US adults had balance disorders, 26% of whom were elderly people (above 65 years) [14]. Approximately 20% of elderly people in the United States have a balance disorder event annually [15]. In fact, dizziness is a common complaint among the elderly population and is a strong predictor of falling events with a negative impact on quality of life [16]. Poor balance is frequently associated with falling [17,18]. In particular, asymmetrical vestibular function may often contribute to falls and fractures in elderly people [19-21].

Balance disorders and consequent falls have progressively represented a burden of disease, accompanied by high costs and pressure on the social services and health care systems related to medical care. This includes repeated consultations, excessive use of diagnostic imaging, and emergency care [22-24]. For example, the first national study in the context of dizziness and vertigo in the Emergency Services of United States of America for 2011 revealed that 25.7% of patient complaints of dizziness and vertigo were associated with balance disorders. The cost was estimated at about US $768 per episode, translating to an annual national cost of US $757 million. In the same context, cardiovascular diseases (linked to 16.5% of these episodes) represented a cost of approximately US $1489 per episode for an annual cost of US $941 million. By comparison, cerebrovascular diseases only accounted for 3.1% of these episodes, but with a cost per episode of approximately US $1059 or an annual cost of US $127 million. With the progressive aging of the population, worsening of this situation is expected in the future [25]. Indeed, vertigo is already contributing to the increasing trend of health care costs, which is linked to the aging of the population [23,24].

In this scenario of global aging, the use of digital solutions has been encouraged. Moreover, the additional pressure of the current COVID-19 pandemic has motivated the broader use of eHealth technologies [26].

#### Digital Health Care and the Elderly

The aging trend represents a relevant challenge to both patients and their families, and to the sustainability of health care systems globally. This is linked to the goal of global health policies for achieving a more active and healthy aging society with autonomy and independence [27,28]. The provision of new health care models, including eHealth services, has been encouraged to tackle access inequities, optimize health outcomes, and ensure autonomy and social support for elderly people. The use of eHealth seems to decrease costs associated with both institutionalization and unnecessary hospital visits [27-29].

eHealth consists of the use of information and communication technologies (ICTs) to support a health care communication channel at a distance, allowing for more efficient delivery of care services with optimized resource allocation. eHealth often contributes to improving the quality of health care services, including faster access to health information, promotion of the globalization of health care, and better health outcomes [30]. The World Health Organization has also recommended eHealth to promote universal health coverage, envisaging higher health care services availability with fewer resources and larger patient interaction. To date, eHealth has been used in the management of many conditions from health literacy promotion to teleconsultations [31]. The remote access systems can actively monitor elderly people in a real-life environment, leveraging the fact that there is an increasing interest and engagement of the elderly with technology. Moreover, eHealth technologies can enhance medical-patient interactions and mitigate many care access inequities. However, digital training of elderly people and caregivers is essential [4,32-34].

#### Assessment and Rehabilitation for the Elderly with Balance Disorders

There are several clinical tests and functional scales, including the Timed Up and Go Test (TUGT), Unipedal Standing Test, and Berg Balance Scale, that allow for assessments of balance, gait, and risk of falling [5,35]. The use of sensors can improve the data quality of these tests and scales [36,37]. Additionally, functional tests of the inner ear, such as videonystagmography or the Video Head Impulse Test, are essential to identify and measure balance disorder cases, including an age-related decline in balance function (prebyvestibulopathy) [6].

Personalized balance training is a relevant option for the treatment of elderly people with balance disorders and risk of falling [5]. This training consists of an exercise-based program to address an individual’s specific balance disorder, with goals of increasing postural stability, improving activities of daily living, and decreasing symptoms. Balance training should be focused on the functional deficiencies identified. Therefore, a prior medical evaluation is necessary to identify the clinical conditions related to poor balance as mentioned previously [38-41]. Moreover, these clinical conditions can affect the outcomes. For example, intervention success is more difficult when the patient has a disorder of both inner ears or has limited mobility due to an osteoarticular disease [38-41]. Exercises delivered through video games can be a promising intervention to achieve greater access and adherence among elderly people [42,43].

Several reviews have addressed the potential of digital solutions to improve the clinical observation and evaluation of balance disorders, and to promote the remote balance rehabilitation of elderly people [36,37,42-47]. However, most of these reviews included studies using a younger population as a preliminary assessment [42,44-47], and the majority did not describe the clinical conditions of the participants that might interfere with the outcomes, especially in the context of balance rehabilitation. Additionally, the clinical applicability of these devices was not assessed [36,37,42-47].

Therefore, there is a gap in this field in terms of evaluating the overall applicability of digital solutions according to the clinical conditions of elderly people with balance disorders and without neurological disease.

### Objectives

The aim of this review was to evaluate and guide the clinical applicability of eHealth devices in the screening, assessment, and treatment of elderly people with balance disorders but without neurological disease.

## Methods

### Design

This systematic review was performed in accordance with the PRISMA (Preferred Reporting Items for Systematic Reviews and Meta-Analysis) statement [48] with the following steps: development of research questions, development of a search strategy with eligibility criteria, data selection, and qualitative analysis.

The protocol for this systematic review was registered in the International Prospective Register of Systematic Reviews (PROSPERO; CRD42019120774) and the complete protocol is available on the National Institute for Health Research program website.

This review focused on answering the following specific research questions, according to the PICO (Population, patient, or problem; Intervention; Control, Comparison, or Comparator; Outcome) strategy [49] (Textbox 1): (1) What are the main contributions of eHealth to elderly people with balance disorders with risk of falling? and (2) Is there any evidence that eHealth improves the quality of health care services in this context? If not, what are the reasons?

**Description of the PICO components.**

- P (Population, Patient, Problem): Elderly people (over 60 years old) with balance disorders and risk of falling; studies with elderly people with functional limitation by neurological disease were excluded
- I (Intervention): eHealth devices for remote health education, screening, assessment, monitoring, or rehabilitation of elderly people with balance disorders with risk of falling
- C (Control, Comparison, Comparator): No intervention, paper booklet information, clinical evaluation, conservative balance training
- O (Outcome): Clinical applicability, increased fall prevention literacy, early identification and evaluation of balance deficits and risk of falling, improved balance and gait performance, reduced rate of falling, increased rehabilitation adherence, increased independence in daily activities

### Definition of Concepts and Keywords used in the Search Strategy

In this study, we defined elderly people as those over 60 years of age [50]. Knudson [51] defined balance as a “person’s ability to control their body position relative to some base of support.” According to Agrawal et al [6], vertigo and dizziness are defined as “sensation of self-motion when no self-motion is occurring or the sensation of distorted self-motion during an otherwise normal head movement” and “sensation of disturbed or impaired spatial orientation without a false or distorted sense of motion,” respectively. Falls refer to “inadvertently landing on the ground, floor or other lower level” [10]. Gait is defined as “the pattern of movement of the body during locomotion” [52].

Telemedicine is defined according to the World Health Organization Group Consultation on Health Telematics [53] as “delivery of health care services using ICT for the exchange of valid information for diagnosis, treatment and prevention of disease and injuries, research and evaluation, and for the continuing education of health care providers.” eHealth is defined according to Eysenbach [54] as:

an emerging field in the intersection of medical informatics, public health and business, referring to health services and information delivered or enhanced through the Internet and related technologies. In a broader sense, the term characterizes not only a technical development, but also a state-of-mind, a way of thinking, an attitude, and a commitment for networked, global thinking, to improve health care locally, regionally, and worldwide by using information and communication technology.

Teleconsultation is defined as “synchronous or asynchronous consultation using ICT to omit geographical and functional distance” [55]. Finally, a sensor is defined as a “device that responds to a physical input of interest with a recordable functionally related output that is usually electrical or optical” [56].

### Search Strategy

Articles were retrieved through searching the PubMed, Google Scholar, Embase, and SciELO databases. The search algorithm included multiple group combinations, as shown in Table 1.

**Table 1:** Search strategy.

Concept	Keywords
Elderly people	(“elderly” OR “older” OR “aged”)
Balance	(“Balance” OR “balance disorder” OR “balance problem” OR “vertigo” OR “dizziness) and/or (“falls” OR “fall detection” OR “fall prevention”) and/or (“gait”)
Telemedicine	(“Telemedicine” OR “eHealth” OR “teleconsultation” OR “technology” OR “sensor”)

### Selection Criteria

The inclusion criteria were randomized controlled trials (RCTs) or quasiexperimental studies (QESs) published in English between January 2015 and December 2019, studies related to use of eHealth in the context of balance and falls, and the sample was restricted to an elderly population (60 years old and above).

The exclusion criteria were: (1) review articles, brief reports, protocols, proof-of-concepts, pilot studies, conference papers, and letters to the editor; (2) studies including elderly people with a reported functional limitation due to a neurological disease; and (3) articles without an age sample reference or with participants aged below 60 years.

### Screening Process and Data Extraction

First, both authors screened the papers independently, looking at titles, abstracts, and methods, and agreed about their inclusion or exclusion according to the eligibility criteria. Second, the potentially relevant papers were retrieved for full-text evaluation against the eligibility criteria. Any articles that were deemed to be questionable in the first stage were included for further evaluation in the second stage. The selection of papers was performed by checking the extracted data and risk of bias.

### Outcome Measures

The main outcomes included population characteristics, balance disorder, identification of faller participants, eHealth platform and services, health benefits, and fall prevention literacy.

### Risk of Bias Assessment

The quality of the evidence to respond to the research questions was independently assessed using the Joanna Briggs Institute (JBI) Critical Appraisal for Experimental Studies and JBI Critical Appraisal Checklist for Quasi-Experimental Studies tools [57]. The two researchers discussed the results of the quality appraisal, reaching a consensus in case of any divergence. The included RCTs were assessed using the Cochrane risk of bias tool [58] to evaluate the risk of internal bias for a series of domains: selection bias, performance bias, detection bias, attrition bias, and reporting bias. Disagreements were solved by consensus between the two researchers.

### Data Analysis

We provide a narrative synthesis of the main outcomes from the included studies. First, the articles were categorized according to the study design. Second, the articles were categorized based on the focus of eHealth services (screening/assessment and treatment/rehabilitation) for comparison of clinical use and applicability according to digital devices.

## Results

### Search Results

A total of 1030 unduplicated articles were identified, 984 of which were excluded after title and abstract screening. Among the 46 full-text publications assessed for eligibility, 25 articles were excluded owing to functional limitations due to neurological disease (n=4), age of participants (n=5), focus on technological implementation (n=2) or model/algorithm (n=8), and specific descriptions of elderly gait parameters (n=6).

**Figure 1:**
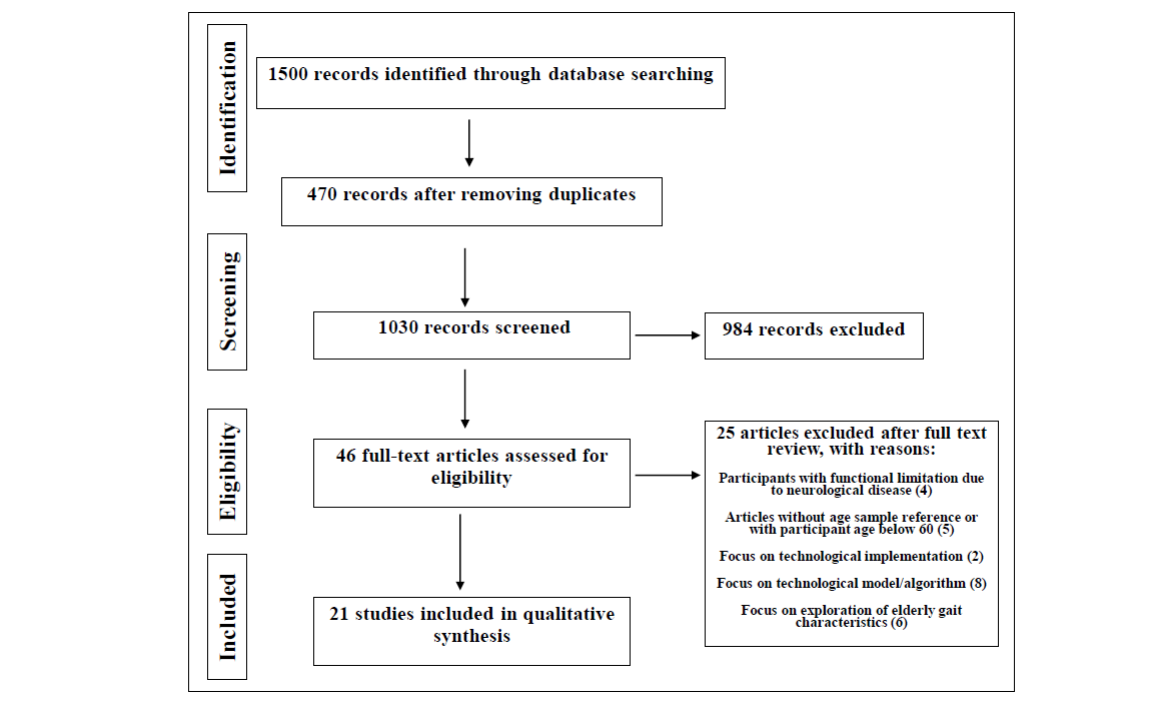
Flow of selection for studies included and excluded in the review.

Twenty-one articles [59-79] were ultimately included in the review (Figure 1).

### Study Design

#### RCT Design

Seven studies were RCTs [59-65], including one cross-over trial without a washout term [64] and one multicenter study [60].

Not all authors clearly described the randomization process [62] and the allocation concealment [62,64] (Tables 2 and 3).

**Figure 2:**
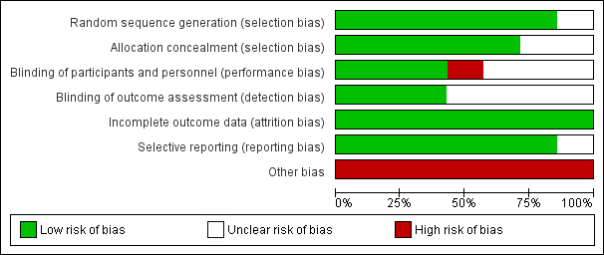
Risk of bias in accordance with the authors' judgment (RevMan version 5.3.).

The inclusion criteria were mentioned in all articles. However, the clinical conditions of the participants were only described in one study [60]. The function of the inner ear was never mentioned. Thus, the expected similarity between the control and intervention groups was not clear. This is relevant because various clinical conditions (eg, cardiovascular, metabolic, inner ear disease, medication) can interfere with the outcomes of balance rehabilitation [38-41]. Therefore, the lack of information about clinical conditions of the participants, including the lack of data about function of the inner ear, was considered as “other bias” and was a common weakness of all included RCTs (Table 3 and Figure 2). This approach led to a worse classification of the quality of these studies.

Additionally, the blinding of participants, personnel, and outcome assessment were unclear in some of these studies.

In the control and intervention groups of all RCTs, a few dropouts for medical and personal reasons were mentioned. However, this was not considered to be sufficiently relevant to have an impact on the results. Only two papers reported intention-to-treat analysis [60,63].

All outcomes were measured in a reliable manner and were considered to have been properly analyzed.

**Table 2:** Methodological quality of randomized controlled trials based on the Joanna Briggs Institute Critical Appraisal Checklist.

Study	Q1^a^	Q2^b^	Q3^c^	Q4^d^	Q5^e^	Q 6^f^	Q7^g^	Q8^h^	Q9^i^	Q10^j^	Q11^k^	Q12^l^	Q13^m^
Eggenberger et al [59]	Y^n^	Y	U^o^	Y	N^p^	U	Y	Y	N	Y	Y	Y	Y
Gschwind et al [60]	Y	Y	U	Y	Y	U	Y	Y	Y	Y	Y	Y	Y
Gschwind et al [61]	Y	Y	U	U	U	Y	Y	Y	N	Y	Y	Y	Y
Lim et al [62]	U	U	U	U	U	U	Y	Y	N	Y	Y	Y	Y
Oesch et al [63]	Y	Y	U	N	N	Y	Y	Y	Y	Y	Y	Y	Y
Ozaki et al [64]	Y	U	U	U	U	U	Y	Y	N	Y	Y	Y	Y
Hong et al [65]	Y	Y	U	Y	Y	Y	Y	Y	N	Y	Y	Y	Y

^a^Question 1: Was true randomization used for assignment of participants to treatment groups?

^b^Question 2: Was allocation to treatment groups concealed?

^c^Question 3: Were treatment groups similar at the baseline?

^d^Question 4: Were participants blind to treatment assignment?

^e^Question 5: Were those delivering treatment blind to treatment assignment?

^f^Question 6: Were outcomes assessors blind to treatment assignment?

^g^Question 7: Were treatment groups treated identically other than the intervention of interest?

^h^Question 8: Was follow-up complete and if not, were differences between groups in terms of their follow-up adequately described and analyzed?

^i^Question 9: Were participants analyzed in the groups to which they were randomized?

^j^Question 10: Were outcomes measured in the same way for treatment groups?

^k^Question 11: Were outcomes measured in a reliable way?

^l^Question 12: Was appropriate statistical analysis used?

^m^Question 13: Was the trial design appropriate, and any deviations from the standard randomized controlled trial design (individual randomization, parallel groups) accounted for in the conduct and analysis of the trial?

^n^Y: Yes.

^o^N: No.

^p^U: Unclear.

**Table 3:** Risk of bias for randomized controlled trials based on the modified Cochrane Collaboration tool.

Study	Selection bias			Other bias		Reporting bias: selective reporting		Performance bias: blinding (participants and personnel)		Detection bias: blinding (outcome assessment)		Attrition bias: incomplete outcome data
	Random sequence generation	Allocation concealment										
Eggenberger et al [59]	L^a^	L	H^b^		U^c^		L		U		L	
Gschwind et al [60]	L	L	H		L		L		U		L	
Gschwind et al [61]	L	L	H		L		U		L		L	
Lim et al [62]	U	U	H		L		U		U		L	
Oesch et al [63]	L	L	H		L		H		L		L	
Ozaki et al [64]	L	U	H		L		U		U		L	
Hong et al [65]	L	L	H		L		L		L		L	

^a^L: low risk.

^b^H: high risk.

^c^U: unclear risk.

#### QES Design

Fourteen studies were QESs [66-79]; only one of these was a multicenter study [73]. Twelve of these studies used the same group of participants [66,68-77,79]. One study used two groups with different participants [67] and another had a control group and an intervention group [78]. However, the expected similarity between the groups was not clear because there was no description of the clinical conditions of the participants, including function of the inner ear. Loss to follow-up was also not mentioned for any of these studies (Table 4).

**Table 4:** Methodological quality of quasiexperimental studies based on the Joanna Briggs Institute Critical Appraisal Checklist.

Study	Q1^a^	Q2^b^	Q3^c^	Q4^d^	Q5^e^	Q6^f^	Q7^g^	Q8^h^	Q9^i^
Zacaria et al [66]	Y^j^	Y	Y	N^k^	Y	Y	Y	Y	Y
Hall et al [67]	Y	U^l^	Y	N	Y	Y	Y	Y	Y
Howcroft et al [68]	Y	Y	Y	N	Y	Y	Y	Y	Y
Lee et al [69]	Y	Y	Y	N	Y	Y	Y	Y	Y
Ponti et al [70]	Y	Y	Y	N	Y	Y	Y	Y	Y
Similä et al [71]	Y	Y	Y	N	Y	Y	Y	Y	Y
Shahzad et al [72]	Y	Y	Y	N	Y	Y	Y	Y	Y
Brodie et al [73]	Y	Y	Y	N	Y	Y	Y	Y	Y
Howcroft et al [74]	Y	Y	Y	N	Y	Y	Y	Y	Y
Chigateri et al [75]	Y	Y	Y	N	Y	Y	Y	Y	Y
Qiu et al [76]	Y	Y	Y	N	Y	Y	Y	Y	Y
Hiesh et al [77]	Y	Y	Y	N	Y	Y	Y	Y	Y
Maneproom et al [78]	Y	U	Y	Y	Y	Y	Y	Y	Y
Nightingale et al [79]	Y	Y	Y	N	Y	Y	Y	Y	Y

^a^Question 1: Is it clear in the study what is the “cause” and what is the “effect” (ie, there is no confusion about which variable comes first)?

^b^Question 2: Were the participants included in any similar comparisons?

^c^Question 3: Were the participants included in any comparisons receiving similar treatment/care other than the exposure or intervention of interest?

^d^Question 4: Was there a control group?

^e^Question 5: Were there multiple measurements of the outcome, both pre and post the intervention/exposure?

^f^Question 6: Was follow-up completed and if not, were differences between groups in terms of their follow-up adequately described and analyzed?

^g^Question 7: Were the outcomes of participants included in any comparisons measured in the same way?

^h^Question 8: Were outcomes measured in a reliable way?

^i^Question 9: Was appropriate statistical analysis used?

^j^Y: yes.

^k^N: no.

^l^U: unclear.

Multiple different measurements of the outcomes were used (Table 4). However, the instrumental inner ear tests were not used as the gold-standard test. This lack of comparison was considered to be a weakness of all of the included QESs. The most commonly applied tests were the TUGT and walking over different distances. One study assessed 1-week daily-life walking [73]. Only one study explored the activities of daily living [75] (Table 5).

The outcomes were considered to have been measured in a reliable manner and were properly analyzed.

**Table 5:** Quasiexperimental studies focused on screening/assessment.

Reference	Population and setting	Participants, N (male/female)	Technology/sensor used (location)	Completed sensor-based procedures	Comparison with fall history or a gold-standard test
Zacaria et al [66]	Hospital	38 (20/18)	WIS^a^ accelerometer and gyrosensor (L2 vertebra)	TUGT^b^ (sensor analysis of performance of each phase)	Classification of faller/nonfaller based on total duration for TUGT completion
Howcroft et al [68]	CDEP^c^	100 (44/56)	Wearable pressure-sensing insoles (pressure sensors-plantar); WIS, 4 triaxial accelerometers (head, pelvis, shanks)	7.62 m walk and 7.62 m walk with cognitive load (ST^d^ and DT^e^ gait)	Classification of faller/nonfaller based on retrospective fall occurrence
Lee et al [69]	CDEP	65 (16/49)	WIS (1 triaxial accelerometer belt around waist, pelvis, sacrum, L3-L5 vertebrae)	TUGT	Short-form BBS^f^ (7 activities), TUGT
Ponti et al [70]	CDEP	36 (11/25)	WIS (1 triaxial accelerometer, waist)	ST TUGT, DT manual TUGT, DT cognitive TUGT	Faller/nonfaller based on retrospective fall occurrence, FES^g^
Similä et al [71]	Senior house/senior physical exercise group	35 (0/35)	WIS (2 accelerometers L3-L5 vertebrae, right hip)	BBS + TUGT + 4 m walk and follow-up after 1 year	Background questionnaire, interview, balance platform assessment with Kinect recording
Shahzad et al [72]	CDEP	23 (7/16)	WIS (1 triaxial accelerometer, L3-L5 vertebrae)	TUGT, STS-5^h^, AST^i^	BBS
Brodie et al [73]	CDEP	96 (39/57)	Pendant sensor 3D accelerometer and barometer (sternum)	1-week daily life walking	Fall history, comparison with TUGT and 10 m walk test
Howcroft et al [74]	CDEP	75 (31/44)	Wearable pressure-sensing insoles (pressure sensor, plantar), WIS 4 triaxial accelerometers (head, pelvis, shanks)	7.62 m walk under ST, 7.62 m walk under DT (verbal-task cognitive load)	Classification of faller/ nonfaller based on prospective fall occurrence
Chigateri et al [75]	Frail elderly people from independent-living retirement homes	23 (6/17)	WIS 1 triaxial accelerometer (L5 vertebra)	TUGT, STS^j^, activities of daily living	Synchronized videos with accelerometer (identification of the beginning of TUGT and of walking episode)
Qiu et al [76]	CDEP/social welfare centers	196 (0/196)	WIS 5 sensors with 3-axis-acceleration, 3-axis angular velocity, 3-axis magnetism each (low back, upper legs, lower legs)	Sensory integration test, limits of stability forward reach, STS-5, TUGT, motor function	Classification of faller based on self-reported history
Hiesh et al [77]	Healthy elderly people	30 (12/18)	Smartphone technology, 1 accelerometer (sternum)	Balance tests standing on a force plate and holding a smartphone against the chest: eyes open/closed DT, semitandem, tandem stance, single-leg stance	Comparison between force plate and smartphone data
Nightingale et al [79]	Local community centers/health care provider offices/senior centers	51 (unknown)	OptoGait system photoelectric technology	10 m walk	TUGT

^a^WIS: wearable inertial sensor.

^b^TUGT: Timed Up and Go Test.

^c^CDEP: community-dwelling elderly people.

^d^ST: single task.

^e^DT: dual task.

^f^BBS: Berg Balance Scale.

^g^FES: Fall Efficacy Scale.

^h^STS-5: Five Times Sit-to-Stand test.

^i^AST: Alternative Step Test.

^j^STS: Sit-to-Stand test.

### Focus of eHealth Services

The 12 QESs were focused on screening and assessment. These studies compared the use of sensors in participants with a history of falling, or with clinical tests and functional scales [66,68-77,79] (Table 5). No instrumental test of the inner ear was performed as a gold-standard test. All RCTs [59-65] and two QESs [67,78] were focused on balance treatment or rehabilitation (Table 6). Again, no instrumental test of the inner ear was used in pre and postrehabilitation assessments.

**Table 6:** Studies focused on treatment/rehabilitation.

Reference	Study type	Setting and population	Participants, N (male/female)	Tested technology	Tested sensor architecture	Sensor-based procedures	Outcome measurements
Eggenberger et al [59]	RCT^a^	CDEP^b^ and retirement homes	71 (25/46): dance group n=24, memory group n=22, control group n=25	VR^c^ video game dancing + Impact Dance Platform treadmill walking + computer screen + training software	VR video game dancing + pressure sensitive platform, treadmill + training software	VR video game dancing with simultaneous cognitive-physical training; treadmill walking with simultaneous verbal memory training; treadmill walking (control)	Gait analysis: ST^d^/DT^e^ 7.3 m walking, Short Physical Performance Battery, fall frequency, 6-minute walk test, measure of fall fear
Gschwind et al [60]	RCT	CDEP	153 (60/93): intervention group n=78, control group n=75	iStoppFalls system: computer + Google TV set top box + Microsoft Kinect + senior mobility monitor + android tablet	Kinect-based system (3D depth sensor), Senior Mobility Monitor (3D accelerometer, barometer)	16-week home-based balance exergames and muscle strength exercises + education booklet (intervention) or education booklet + usual activities (control)	Estimated risk of falling; mobility, self-care, usual activities, pain, discomfort, anxiety, depression, health questionnaires, cognitive performance, walking task, STS-5^f^, TUGT^g^, technology use
Gschwind et al [61]	RCT	CDEP	124 (42/82): step-mat training group n=39, Microsoft-Kinect group n=24, control group n=61	Input device, computer, USB modem, TV, exergames, Microsoft Kinect or electronic mat	Pressure-sensitive electronic mat, Kinect-based system (3D depth sensor)	Unsupervised 16-week home exercise using exergames or educational booklet about evidence-based health and fall prevention advice + usual activities (control)	Risk of falling, health and disability measure, STS-5, TUGT, cognitive performance
Hall et al [67]	QES^h^	CDEP	16 (0/16): group A n=8, group B n=8	Nintendo Wii Fit System: computer interface + monitor + Wii balance board + games Ski Slalom/ Table Tilt	*Balance* Board: force platform	Wii Fit balance test + games, followed by SOT^i^ and LOS^j^ test, CDP^k^ (group A); SOT and LOS test, CDP, followed by Wii Fit balance test + games (group B)	Dynamic Gait Index, TUGT, gait speed
Lim et al [62]	RCT	CDEP	36 (11/25): intervention group n=18, control group n=18.	Wearable balance biofeedback (system (vibrotactile, auditory and visual biofeedback)	Biofeedback headband: 8 vibrotactile actuators, 2 bone-conducting acoustic transducers, 3 light-emitting diodes; and gyroscopes (lower back)	2-week training with real-time multimodal biofeedback of trunk sway or 2-week training without biofeedback (control)	Standing:1 leg (eyes open), feet together, firm surface and foam, tandem stance (EC^l^); self-paced 8 m walking (EC); 8 m walking with head turning; 8 tandem steps (EC)
Oesch et al [63]	RCT	Geriatric rehabilitation center	54 (29/25): intervention group n=26, control group n=28	Windows Kinect (exergame)	Kinect-based system (3D depth sensor)	10-day self-regulated training with exergames or 10-day self-regulated conventional training with instruction leaflets (control)	Adherence, motivation, enjoyment, sensor-based walking test
Ozaki et al [64]	RCT	Prefrail or frail CDEP	27(7/20): intervention group n=14, control group n=13	BEAR^m^ system: Stand-and-ride transport robot + wearable helmet and suspending device + software	Stand-and-ride transport robot with two inverted wheel motors	6-week based BEAR training first group or 6-week based conventional balance training first group (control)	Gait speed, tandem gait speed, functional reach test, TUGT
Hong et al [65]	RCT	CDEP	23 (0/23): intervention group n=10, control group n=13	tablet, web app, signaling server module network address translator traversal module	Web Real-Time Communication (WebRTC) technology	12-week telepresence exercise sessions or maintained lifestyle (control)	Senior fitness test, BBS^n^, fall-related self-efficacy, FES^o^, Fear of falling questionnaire
Maneproom et al [78]	QES	Senior housing	64 (13/51): intervention group n=32, control group n=32	robot, robot-installed fall prevention software	8-inch touchscreen installed at robot head	robot-installed fall prevention software + personal coaching + fall prevention handbook or fall prevention handbook only (control)	TUGT, BBS, fall prevention questionnaire

^a^RCT: randomized controlled trial.

^b^CDEP: community-dwelling elderly people.

^c^VR: virtual reality.

^d^ST: single task.

^e^DT: dual task.

^f^STS-5: Five Times Sit-to-Stand test.

^g^TUGT: Timed Up and Go Test

^h^QES: quasiexperimental study.

^i^SOT: Sensory Organization Test.

^j^LOS: Limits of Stability.

^k^CDP: computerized dynamic posturography.

^l^EC: eyes closed.

^m^BEAR: Balance Exercise Assist Robot.

^n^BBS: Berg Balance Scale

^o^FES: Fall Efficacy Scale.

### Population Characteristics

The included studies had large differences in sample sizes, ranging from 23 to 153 participants [59-65] among RCTs and from 16 to 196 participants [66-79] among QESs. As shown in Tables 5 and 6, many studies included a small sample size that was described as a limitation.

The age range was 60-91 years for the RCTs and 60-92 years for the QESs. Most of the studies included more women than men. In four studies, only women participated [65,67,71,76]. The decision to only recruit women was explained in one study as “to avoid the influence of gender differences on risk of falling” [76]. Two studies excluded the few male participants [67,71] and the remaining article did not describe the reason for the exclusive participation of women [65]. One study did not describe the age range or the gender distribution of the participants [79].

The participants (≥60 years old) were recruited from the community [60-62,64,65,67-70,72-74,76,77], gerontology services [71,75,78], both [59,79], or at a hospital [66]. One study included participants who were referred for geriatric inpatient rehabilitation [63].

Most of the studies did not describe the characteristics of health conditions of the sample [59,62,64-66,68-72,74-77,79]. Only some authors provided quantitative data about the participants’ medication use [60,61,73,78] and their comorbidities [60,61,63,67,73,78]. One study [60] highlighted the following comorbidities of the participants: heart problems, high blood pressure, osteoporosis, lower back pain, hip pain, knee and/or leg pain, and foot pain. Two studies excluded participants with self-reported balance disorders [62,63]. Two other studies included frail or prefrail elderly adults [64,75].

### Balance Disorder and Identification of Faller Participants

The included studies used functional balance tests, with or without sensors, to evaluate balance and risk of falling. An objective identification via exploration and quantification of the function of the inner ear by instrumental tests was not employed in any of the considered studies, as mentioned above. Therefore, the presence of prebyvestibulopathy or other balance disorders was not known.

Some authors highlighted the potential of sensor-based tests in identifying early balance deficits [71] and in evaluating the risk of falling [66,72,73,76,77]. Improved balance and gait with technology-based training were mentioned in some studies [59,62,64].

The identification of faller participants based on retrospective [68,70,73,76] or prospective occurrence of falling [74] was employed to compare the technology results. The benefits of virtual training in reducing the risk of falling was also described [60,61,65,78].

No study focusing on detection of falling fully complied with the inclusion criteria of this review (RCTs or QESs, published in English between January 2015 and December 2019, restricted to the population 60 years or older).

### eHealth Platform and Services

Different platforms were used for the provision of eHealth services. The main platforms identified were computer-based apps, either via the internet or mobile based platforms (Tables 5 and 6).

As mentioned above, 12 studies focused on screening or assessment [66,68-77,79] using different types (wearable inertial, wearable pressure, pendant, smartphone), quantities (range 1-5), and locations (head, sternum, lumbar vertebra, pelvis, hip, leg, shanks, foot) of sensors. Single or combined sensor-based tasks were employed. Only two studies [73,75] evaluated activities in a real-life environment (Table 5).

Nine studies explored balance rehabilitation [59-65,67,78] with different exercises and duration of training. The follow-up time was short (less than 6 months) in most of the studies, with the longest follow-up of 1 year [59]. The development of eHealth services was explored both inside and outside the laboratory environment (Table 6).

One study used a robot to provide information about training and fall prevention. However, the authors pointed out that the screen and the volume speaker were not adequate for use by elderly people [78].

The use of technical language and the presence of disabilities such as visual and hearing impairment were highlighted as the main barriers in using eHealth [78].

### Health Benefits

Only one study did not report better adherence, enjoyment, motivation, and balance performance with virtual training. This was explained by the possible fragility of the sample included in the study and by the short duration of the training intervention [63].

The remaining papers emphasized the potential contribution of digital solutions to improve balance performance and risk of falling. The sensors used during balance tests improved the evaluation of balance and gait [66,68,69,71,72,79] and improved the identification of potential faller participants [70,73-77]. In addition, the use of eHealth devices for balance rehabilitation increased balance and gait performance [59,60,62,64,65,78], and reduced the risk of falling [60,61]. However, no long-term follow-up was reported. Virtual programs of falls prevention seemed to increase knowledge on the subject [78] (Table 7).

**Table 7:** Health benefits: conclusions from all studies.

Reference	Study type	Conclusions
Zacaria et al [66]	QES^a^, screening	Single wearable sensor during TUGT^b^: an improved tool in evaluating fall risk
Howcroft et al [68]	QES, screening	Sensor-based gait assessment: potential of identification of gait changes
Lee et al [69]	QES, screening	Advantages of wearable sensor as an outside laboratory tool
Ponti et al [70]	QES, screening	Improved potential of identification of fallers with single sensor-based DT^c^ TUGT
Similä et al [71]	QES, screening	Sensor-based walk test: a screening tool to identify early signs of balance deficits
Shahzad et al [72]	QES, screening	Importance of sensor-based TUGT, STS-5^d^, and AST^e^ on fall risk estimation
Brodie et al [73]	QES, multicenter screening	Better sensor-based daily-life gait assessment to discriminate fallers
Howcroft et al [74]	QES, screening	Sensor: potential to discriminate differences between ST^f^ and DT gait and between prospective fallers and nonfallers
Chigateri et al [75]	QES, screening	Wearable accelerometer: useful for nonsedentary activity recognition and gait detection in frail older adults outside lab facilities
Qiu et al [76]	QES, screening	Potential use of wearable inertial sensor-based systems for elderly fall risk assessment
Hiesh et al [77]	QES, screening	Validity of smartphone for evaluation of postural stability and fall risk stratification in older adults
Nightingale et al [79]	QES, screening	Using Optogait system: TUGT as a tool for screening balance deficits
Eggenberger et al [59]	RCT^g^, rehabilitation	Virtual reality game dancing with simultaneous cognitive-physical training and treadmill walking with simultaneous verbal memory training: potential to enhance gait variables
Gschwind et al [60]	RCT, rehabilitation	iStoppFalls program reduced physiological fall risk and improved postural sway
Gschwind et al [61]	RCT, rehabilitation	Step-mat-training and Microsoft-Kinect exergames reduced fall risks, Step-mat-training improved specific cognitive functions; neither intervention improved balance control
Hall et al [67]	QES, rehabilitation	WiiFit feasible to safely use, Ski Slalom game similar effect as computerized dynamic posturography
Lim et al [62]	RCT, rehabilitation	Balance training with biofeedback: most beneficial for the most difficult tasks but with few long-term benefits
Oesch et al [63]	RCT, rehabilitation	Superior results of conventional training with respect to adherence, enjoyment, and motivation; no difference of balance during walking between conventional and training with exergames
Ozaki et al [64]	Crossover trial without a washout term, rehabilitation	BEAR^h^ training more effective for improving dynamic balance and lower extremity muscle strength
Hong et al [65]	RCT, rehabilitation	Telepresence exercise program: effective to improve balance and reduce fear of fall; no significant difference of fall efficacy between intervention (telepresence exercise sessions) and control group (maintained lifestyle)
Maneproom et al [78]	QES, rehabilitation	Robotic fall prevention program increased fall prevention knowledge, promoted exercises, and improved balance

^a^QES: quasiexperimental study.

^b^TUGT: Timed Up and Go Test.

^c^DT: dual task.

^d^STS-5: Five Times Sit-to-Stand test.

^e^AST: Alternative Step Test.

^f^ST: single task.

^g^RCT: randomized controlled trial.

^h^BEAR: Balance Exercise Assist Robot.

### Fall Prevention Literacy

None of the studies explored the previous health literacy of the participants. Only two papers described the educational level of the participants [60,78].

One study compared use of a fall prevention software to a conventional handbook to evaluate the improvement of knowledge on fall prevention. Both the intervention and control groups showed improvement in knowledge, without a significant difference [78].

## Discussion

### Principal Findings

Population aging, and the associated vulnerability to the development of multiple chronic pathologies and balance disorders, have motivated research and the implementation of new strategies for the provision of health care. eHealth devices have been studied to help assess balance and gait performance, risk of falling in and outside a laboratory setting, and to perform in-home balance rehabilitation. In this review, we confirmed the potential of eHealth to complement the health care of elderly people. However, most of these studies were not designed to provide clinical guidelines.

Despite growing interest about this subject in the last 20 years, we decided to focus on studies published in the last 5 years (RCTs and QESs), taking into consideration both continuous advances in technological innovation and the opportunity to apply new clinical applications in balance disorder and risk of falling for the elderly population.

Unlike other reviews, our eligibility criteria ruled out many initially retrieved articles, especially studies with participants under 60 years old, those without reporting the age of participants, or with participants having a functional limitation due to neurologic disease. Therefore, only 21 articles fully complied with the requirements of this review [59-79].

Except for one study [63], the others showed the potential of eHealth to evaluate balance assessment and risk of falling of elderly people and to promote balance training. The eHealth devices allowed collecting additional information about the balance, gait, and risk of falling of elderly people, and to monitor their daily activities.

In particular, eHealth seems to provide an opportunity for increasing medical-patient interactions and to reduce access inequities [30]. In 1996, Viierre et al [80] had already mentioned the potential of eHealth in this field: “remote medical diagnosis and treatment facilities could make the few vestibular disorder specialists much more available to patients.” However, as observed in other reviews [36,37,42-47], the differences in methodologies and of variables included in the studies did not allow for a proper comparison to guide clinical applicability.

First, there was a broad range of sample sizes, which were generally quite small (ranging from 16 to 196 participants). A small sample of participants is considered a limitation for extrapolating the results, especially for the exploration of risk of falling.

Second, there were missing data about the clinical conditions of the participants. Except for one study [60], several volunteers were recruited from the community and were defined as “healthy” elderly people only based on a self-reported assessment. There were also participants recruited from geriatric services without reference to their clinical conditions. Despite the exclusion of participants with self-reported balance disorders in two studies [62,63], we consider that the exclusion rules should be more rigorous and based on objective data such as instrumental inner ear tests. We have to take into consideration that elderly people can have instability due to many conditions, including the normative aging process, and therefore the outcomes from a balance rehabilitation intervention could be sensitive to these differences [38-41].

Third, different research methodologies were used for screening and assessment. We observed a wide range of clinical tests, functional scales, faller classifications, and sensor-based tasks among the included studies. The lack of homogeneity of these variables limited an appropriate comparison among the studies. Moreover, functional inner ear tests were not used as the gold-standard test. We consider this as a weakness common to all studies.

Fourth, different types of sensors were used for screening and assessment. Similar to the findings of other reviews [36,37,44-47], the studies employed mainly accelerometers, with variations in both number and body location.

Fifth, as observed previously [42,43], studies focusing on treatment and rehabilitation used different devices, training durations, and follow-up times. Some authors employed supervised training. In one study, this was used a telepresence-based exercise platform [65]. Others employed in-home self-regulated exercises training [61], thereby avoiding the need for participants to travel to the rehabilitation center. None of the studies described pre and postintervention data about the function of the inner ear. The studies did not verify the long-term effect of training, especially with respect to fall occurrence. Only two studies explored a sensor used in real-life activities [73,75], which is relevant since it allowed for a better evaluation of the remote interaction and monitoring of daily activities.

Additionally, we observed a constraint related to the use of devices that are not fully adequate to match the abilities of elderly people [78]. We also highlight the importance of providing a better definition of the eHealth user profile to improve adherence.

Future studies in this field should consider the above topics as a starting point, as well as for health policy implementations on eHealth apps for elderly people with balance disorders.

The use of eHealth can play an important role as a complementary method to provide health care services, encouraging health promotion and patient participation, as well as allowing for the remote management of balance disorders.

### Recommendations

Based on this review, we can provide the following recommendations to improve studies and applications of eHealth for preventing fall risk in the elderly population.

First, this review highlights the need for further research on the use of eHealth devices in proper clinical settings. This represents an opportunity to be explored, reaching out to elderly people with balance and risk of falling.

Second, despite several efforts to explore balance among the elderly, there is still a need for better characterization and description of the health condition of the population under study. In particular, we recommend future studies to include the results of functional tests of the inner ear as a gold-standard test or for comparison of the outcome before and after remote balance rehabilitation. Most of the interventions were developed with only functional balance tests. Future studies should also focus on the real-life environment, allowing for additional information of the daily activities among elderly participants.

Third, a longer follow-up time is important to evaluate the long-term benefits of eHealth tools on the balance performance and risk of falling of elderly people.

Finally, the eHealth devices should be user-friendly to improve adherence among elderly people.

### Limitations

This review was limited to articles written in the English language and available on the PubMed, Google Scholar, Embase, and SciELO databases for the last 5 years; therefore, it is possible that relevant studies were missed.

### Conclusions

The inclusion of eHealth services can play a critical role for the better provision of health care to elderly people with a balance disorder and risk of falling. The differences in populations, methodologies, eHealth devices, and follow-up times of the included studies did not allow for a clear comparison between results, therefore limiting the possibility of obtaining valid guidance for clinical applicability. More rigorous studies are recommended.

## Abbreviations

ICT: information and communication technologies
JBI: Joanna Briggs Institute
QES: quasiexperimental study
RCT: randomized controlled trial
TUGT: Timed Up and Go Test
